# Age of onset of self-harm in children and adolescents: a scoping review

**DOI:** 10.1186/s13034-025-00982-6

**Published:** 2025-11-19

**Authors:** Daisy Wiggin, Doireann Ní Dhálaigh, Elaine McMahon, Fiona McNicholas, Eve Griffin

**Affiliations:** 1 School of Public Health , University College Cork , Cork , Ireland; 2 National Suicide Research Foundation , Cork, Ireland; 3 Department of Child and Adolescent Psychiatry, School of Medicine, University College Dublin, Dublin, Ireland; 4 Department of Paediatric Liaison Psychiatry, CHI, Crumlin, Dublin, Ireland; 5 Lucena Child and Adolescent Mental Health Services , St John of God, Dublin, Ireland

**Keywords:** Self-harm, Suicidal behaviour, Non-suicidal self-injury, Age of onset, Adolescents

## Abstract

**Background:**

Self-harm is associated with significant distress in children and adolescents. The objective of this scoping review was to map the age of onset of self-harm in people aged ≤ 18 years alongside the definitions, operationalisation, and research methods used to determine onset.

**Method:**

Following JBI guidance, this review included studies reporting the age of onset of self-harm in people aged ≤ 18 years in any context. Medline, PsycInfo, Embase, CINAHL Plus, and Web of Science were searched last on 7th May 2025 and supplemented by a grey literature search. Data were subject to basic coding and narrative and graphical presentation.

**Results:**

A total of 42 studies were included in the review. Age of onset ranged from 9 to 18 years, but most studies reported a mean age between 12 and 14 years. The majority of studies defined self-harm as either suicidal or non-suicidal (85%), with non-suicidal self-harm distributed in favour of a slightly younger onset age. Studies with a younger sample tended to report a younger age of onset. Most studies used cross-sectional methods (81%) and retrospective report (71%) to capture onset age.

**Conclusions:**

Earlier age of onset is associated with the use of multiple methods of self-harm, self-harm of longer duration and increased frequency. A clear understanding of age of onset of self-harm is necessary to inform clinically relevant research and the timely targeting of developmentally prevention and early intervention strategies.

**Supplementary Information:**

The online version contains supplementary material available at 10.1186/s13034-025-00982-6.

## Introduction

Rates of self-harm are higher among children and adolescents compared to older groups [1]. Self-harm is associated with an increased risk of dying by suicide in children and adolescents [2–4], making it a critical target for suicide prevention. Concerningly, rates of self-harm have been rising in children and younger adolescents in recent times [1, 5–7], providing reason to believe that the age of onset of self-harm is decreasing [8]. A growing evidence base implicates the age of onset of self-harm in its future course. An earlier starting age of self-harm is associated with more frequent acts [9, 10], using multiple methods, being more likely to attend hospital [11, 12], and engaging in self-harm for a longer duration [10]. Developing prevention and intervention strategies is imperative to reduce self-harm and associated distress. A nuanced understanding of the age of onset of self-harm in children and adolescents is critical to ensuring that these strategies are developmentally appropriate.

Age of onset is often studied in the context of medical and psychiatric diagnoses. Determining the age of onset of mental disorders (referred to as emotional or psychological distress henceforth; [13]) can be difficult [14]. This has resulted in a multitude of operationalisations (turning an abstract concept into a measurable construct) of onset of psychological distress in the literature, namely first complaint, first diagnosis, first hospitalisation, and first contact with intervention [15]. Self-harm is often discussed in relation to psychiatric symptoms and diagnoses. There is a dearth of literature mapping how the onset of self-harm is operationalised and discussing how this might impact the estimates reported. Some studies employ retrospective report and use validated questionnaires to assess onset [9, 11, 12], and others collect information from self-harm presentations to hospital [16], with the first presentation potentially being operationalised as onset. This variability in approaches contributes to the difficulties in pooling obtained onset estimates across studies and potentially obscures accurate estimates.

Two prior reviews have synthesised age of onset of NSSI and self-harm. One review reported an age of onset of NSSI between 12 and 14 years [17] and the other an average of 12.81 years (95% CI 11.78–13.84) for self-harm [18]. These reviews were not solely focused on age of onset, and so the operationalisations of and methods used to determine onset in the included studies are not discussed in detail. This, alongside study design, variation in nomenclature used [19], study settings, and methodological differences obfuscates a clear understanding of the age of onset of self-harm. In addition to capturing age of onset of self-harm, the current review intended to report and highlight differences in definitions, operationalisations, and research methods used in the measure of self-harm age of onset; this nuance has been missed in prior reviews.

A scoping review is an appropriate study design to map the age of onset literature with respect to the heterogeneity discussed [20]; in this case, to improve our understanding of how the age of onset of self-harm is operationalised, measured, and defined, and to identify when this has been robustly achieved to inform future research. The aims of this review were to (1) capture the age of onset of self-harm in people aged ≤ 18 years and (2) map this alongside the definitions, operationalisation, and research methods used to determine onset.

## Methods

This scoping review followed JBI guidance, employing the Population, Concept(s), and Context approach [21] and is reported in accordance with PRISMA-ScR guidelines [22] (Supplementary file 1). A protocol was published prior to the conduct of this review [23]. During the screening processes, minor amendments were made to this protocol (described in detail in Supplementary file 2).

### Eligibility criteria

The population of interest was young people who were aged ≤ 18 years, or people 19 years or older who were exclusively reporting on self-harm first experienced aged ≤ 18 years (see Supplementary file 2 for amendment rationale). While adolescence has been argued to last until 25 years of age [24], this age group was chosen due to the widespread application of 18 as the transition age from child and adolescent mental health services to adult services in a variety of countries [25].

The concepts of interest were self-harm and the age at which a young person engages in self-harm for the first time, often regarded as the age of onset. Given the diversity of definitions of self-harm in the literature, the intention was to capture any self-harm definition, most of which fall under the remit of the following definition: ‘an act with a non-fatal outcome in which an individual deliberately initiates a non-habitual behaviour that without intervention from others, will cause self-harm, or deliberately ingests a substance in excess of the prescribed or generally recognised therapeutic dosage, and which is aimed at realising changes that the person desires via the actual or expected physical consequences’ [13]. This definition does not divide self-harm behaviours based on motive(s) or level of suicidal intent and is inclusive of suicide attempt and non-suicidal self-injury (NSSI) for example. There is evidence that the intention of a self-harm act can change while the act is taking place [14] and that there may be ‘non-suicidal’ reasons (e.g., relief from terrible state of mind) and a wish to die co-occurring in the same self-harm act [15]. This makes meaningfully capturing intent in a category or definition of self-harm challenging.

The concept of the onset of self-harm can depend on the context. Drawing on mental health research, onset has been operationalised as first presentation to hospital, first complaint, or first diagnosis [15]. The intention was to be inclusive of all contexts where self-harm can occur or present. Such contexts might include hospital-presenting self-harm, self-harm that comes to the awareness of a parent, teacher, or general practitioner, and self-harm that is disclosed during participation in research. Capturing the context offers exploratory insight for professionals in these settings into the age of onset of self-harm across such settings, contexts, and research methodologies.

The sources of evidence in this review were primary research studies. Study protocols, opinions, letters to the editor, case reports, among others, were not deemed useful to meet the objectives of this review. Detailed eligibility criteria are outlined in Table 1 (see Supplementary file 2 for amendments).

**Table 1:** Age of onset scoping review eligibility criteria

Inclusion criteria	Exclusion criteria
Onset age (e.g., first presentation, first disclosure, etc.) is reported as a mean/median/mode OR age of onset is split to early/late and is confined to 0–18, the late/early categories must be delineated as (for example) under 12 and 13–18	Age of onset is not reported as a measure of central tendency (e.g., mean/median/mode)
The current sample is people who are aged up to and including 18 OR the current sample includes people 18+, BUT the focus of the paper is onset of self-harm in people aged up to and including 18	The sample includes people who are 19 years of age or older and the focus of the study is not onset in the childhood/adolescent period as defined for this review (0–18 years)
The sample is parents, health or social care professionals, or teachers reporting on a direct disclosure or perceived first act of self-harm by a person known to them	The study sample is comprised of people reporting their general attitudes and/or beliefs about the age of onset of self-harm
Any self-harm term or definition used by authors	Study examines self-harm outside the scope of the review, for example, behaviours more commonly observed after experiencing injuries or other medical diagnoses, such as intellectual disabilities or autism (these behaviours are often intense, repetitive, rhythmic behaviours such as eyeball pressing and head banging)
Any measures used to assess age of onset	The sample spans 0–18 and 18 + and onset age is not separately reported for each group
Any context (e.g., health-service presenting, community, school, third sector)	Study reports on thoughts of self-harm and/or thoughts of suicide only
	Study protocols, editorials, letters to the editor, case reports, case series, all types of literature reviews
	Duplicate records
	Full text not available

### Search strategy

**Fig. 1:**
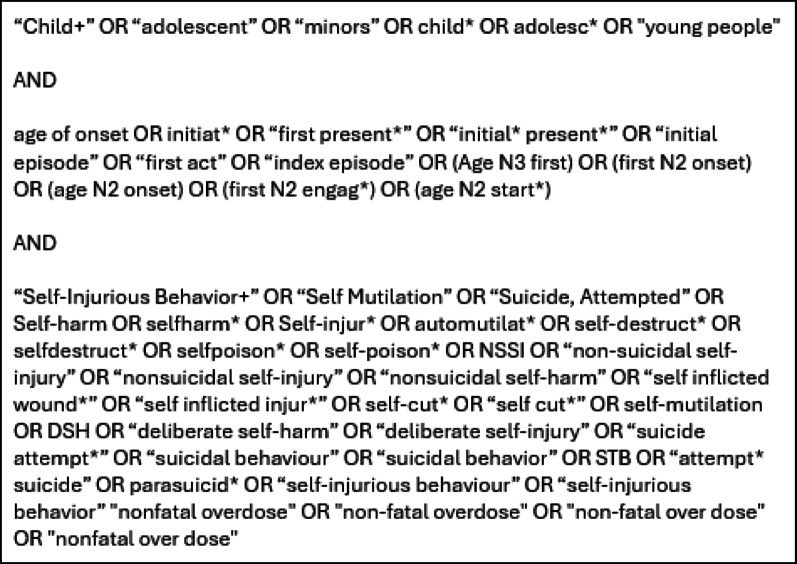
Search strategy for medline

Electronic searches were conducted in Medline (EBSCO), PsycInfo (EBSCO), Embase (Elsevier), CINAHL Plus (EBSCO), and Web of Science (Clarivate) from inception to 26th June 2023, and were last updated on 7th May 2025. The search strategy was designed by DW in consultation with prior systematic reviews on self-harm and a university librarian. It involved terms relating to “young person,” “age of onset,” and “self-harm” (Fig. 1). The search strategies used in the above databases and the grey literature can be found on the Open Science Framework (OSF) registration [26].

Grey literature sources included the first 10 pages of results from a Google Scholar search and a search in BASE (https://www.base-search.net/) conducted on 9th November 2023. The grey literature further included screening titles of reports from the World Health Organization’s suicide and suicide prevention publications, the mental health conditions and paediatric conditions of the Agency for Healthcare Research and Quality website, and the publication sections of the following charity and third-sector organisation’s websites: the Health Service Executive’s Connecting for Life, the National Suicide Research Foundation, Jigsaw, the Samaritans, and the Mental Health Foundation. There were no time or language limits applied to the search. Reference lists of records included after full-text review were screened, which was an amendment to the protocol (Supplementary file 2).

### Study screening and selection

Records resulting from the database and grey literature searches were imported into Zotero, where duplicates were automatically detected but hand removed. The resulting records were then imported into a review set up in Rayyan (www.rayyan.ai) for title and abstract screening.

Prior to commencing screening and selection, two reviewers (DW and DND) piloted the eligibility criteria independently to ensure comprehension and utility of the criteria. Both phases of screening, title and abstract and full text, were conducted by two reviewers independently who met regularly to resolve conflicts in selection decisions. Some conflicts were discussed with EMcM, EG and FMcN to come to a resolution.

An initial scope of the literature prior to undertaking the review revealed many instances where age of onset was reported but was not a primary focus of the study and therefore not mentioned in the title or abstract. To capture as much literature as possible, studies were forwarded to full text screening if title or abstracts mentioned: prospectively measuring onset of self-harm in the eligible age group, retrospective collection of information on the onset, initiation, development of, starting, first presentation, index presentation of self-harm. A similar approach was used in a prior scoping review [27].

Study authors were contacted when full texts of records deemed relevant at title and abstract screening stage were not accessible. Full texts were not available for 30 articles, the primary reasons for this were no responses from study authors and full reports not being available from published abstracts. Any records whose full texts were not in English were translated using DeepL (https://www.deepl.com/translator) to assess eligibility for inclusion.

### Data extraction and quality assessment

The data extraction form was piloted by DW and DND independently. Proposed changes were discussed with the wider team, amendments from the protocol are presented in Supplementary file 2. This guidance document is available on the OSF registration [26]. Data extraction was performed by DW and independently checked by DND against the guidance document.

Included studies were assessed for quality using the Quality Assessment with Diverse Studies (QuADS) tool by DW [28]. This has been done in prior scoping reviews in the area of self-harm [29] and improves the utility of review findings for future research and practice [30]. The QuADS tool was selected as it is suited to assessing a variety of study designs.

### Data collation and presentation of results

Several data items were coded after data extraction was completed. These included study design, study setting, self-harm definitions, and methods used to assess the age of onset. Study design was coded into cross-sectional, longitudinal, retrospective, retrospective cohort study, or randomised controlled trial (RCT). Study setting was coded into school, hospital emergency department, hospital inpatient, outpatient, community, and mixed. Self-harm definitions were coded according to whether they were inclusive of any intent or motivation, excluded or included certain intents or motivations, or did not mention the intent or motivation of the act.

The findings are presented in narrative format. Descriptive information in the form of proportions (%), and measures of central tendency are reported. The ages of onset extracted from included studies were collated in groups according to certain themes of interest, including self-harm definitions, sample characteristics, and clinical characteristics. While an objective of the review was to present age of onset alongside operationalisation of onset, most of the included studies used the same operationalisation (retrospective self-report), so presenting the results across differing onset operationalisations was not deemed to be informative. The narrative findings are supported by graphs and tables.

## Results

**Fig. 2:**
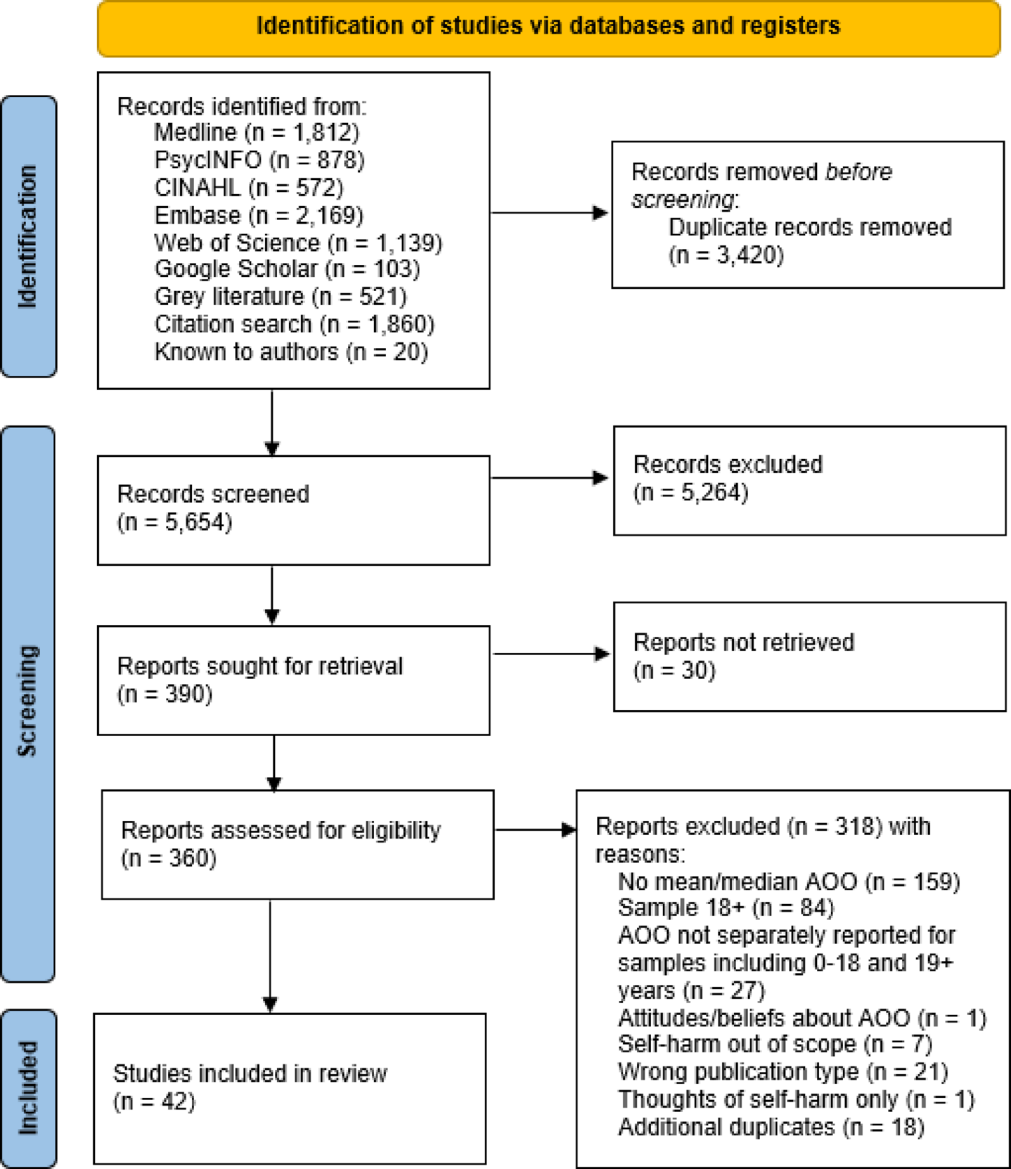
PRISMA flow diagram; AOO, age of onset

A total of 9074 records were identified from all sources, of which 3420 were duplicates, leaving 5654 titles and abstracts to be screened. After this, 390 full texts were sought, and 360 were accessible and thus screened. Full text screening resulted in 318 records being excluded and 42 records included in the review (Fig. 2). Reasons for excluding records during the full text screening phase are described in Supplementary file 3. Of the 42 included studies, 36 were identified by database and grey literature searches, three were identified from the reference lists of included studies [31–33], and three studies were known to the review team [34–36].

### Characteristics of included studies

Key characteristics of the included studies are summarised in Table 2. Age of onset is reported for *n* = 12,392 participants across 41 included studies; one study provided inconsistent reports of sample size. A large proportion of studies were conducted in the United States (38.1% [32, 35–48]), three studies each were conducted in China [49–51] and Australia [52–54]; two studies each in France [55, 56], Canada [57, 58], England [59, 60], and Spain [34, 61]; and one each in Czech Republic [62], Germany [63], Hungary [31], Iran [64], Mexico [65], Norway [9], Peru [66], Poland [67], Serbia [68], Sweden [33], Taiwan [69], Turkey [70]. Most studies (83.3%) were published after 2010. The funding source was reported in 71.4% of studies. Two thirds of the studies were cross-sectional in overall design. Self-harm onset was part of the study aims, objectives or hypotheses in 37.2% of studies. In 52.4% of cases, age of onset was reported as per study defined categories or outcomes such as gender, type of self-harm, and trajectory.

The samples in 88.1% of the studies were children and adolescents, two studies included parents reporting on their child’s self-harm, and three included adults reporting on self-harm they experienced as an adolescent. A slight majority of studies (52.4%) were conducted in clinical hospital inpatient or outpatient settings, 33.3% were conducted in the school or community setting, and the remainder took place in a mixture of settings. Clinical samples, comprised of people who had been given a psychiatric diagnosis, accounted for 54.8% of the included studies. Just over half (57.1%) of the studies collected data on the methods of self-harm and just 7.1% reported method of onset act separately from subsequent acts.

**Table 2:** Characteristics of included studies in the age of onset of self-harm scoping review

Author	Country	Study design	Self-harm term and definition	Onset operationalisation	Sample size (self-harm subgroup, if applicable)	Study setting	Sample age	Age of onset in years (range, if reported)
Abram et al., 2008	USA	Cross-sectional	SA: Not specified	Retrospective self-report	*N* = 1,172 (11%)	Forensic	10–18 years	Mean 12.7 (5–17)
Albores-Gallo et al., 2014	Mexico	Cross-sectional	NSSI: as per DSM-5 criteria and as per the question ‘Do you hurt yourself without intending to kill yourself?’	Retrospective self-report	*N* = 533 (inconsistently reported)	School	11–17 years	Mean 11.9 (6–15) Males: 11.9 (8–15); Females: 11.9 (6–15)
Andrews et al., 2013	Australia	Prospective longitudinal	NSSI: Direct, deliberate and socially unacceptable destruction of one’s body tissue without conscious suicidal intent	Retrospective self-report	*N* = 2,640 (6%)	School	12–18 years	Mean NSSI Continuation: 12.3 (6–15); NSSI Cessation: 12.9 (ns)
Aouidad et al., 2020	France	Cross-sectional	SA: Not specified	Not specified	*N* = 302	Hospital inpatient	11–17 years	Mean Non-BPD-Single attempt: 14.7 (ns); BPD-Single attempt: 14.6 (ns); BPD- Multiple attempt: 14.1 (ns)
Bennett et al., 2011	USA	Retrospective chart review	Self-embedding: Self-injurious behaviour involving the insertion of inanimate objects into the soft tissues, either under the skin or into muscle	Not specified	*N* = 11	Hospital	14–18 years	Mean 16 (14–18)
Brager-Larsen et al., 2022	Norway	Cross-sectional	SH: intentional poisoning or self-injury, regardless of intention to die	Retrospective self-report	*N* = 103	Outpatient	12–18 years	Median 13.2 (4–17)
Brezo et al., 2007	Canada	Prospective longitudinal	SA: Not specified	Parental or self-report	*N* = 4,488 (2%)	School	15–18 years	Median 13.0 (ns)
Buresova et al., 2015	Czech Republic	Cross-sectional	SH: Culturally unacceptable deliberate injury inflicted on one’s own body without a conscious suicidal attempt	Retrospective self-report	*N* = 1,026 (18%)	Not specified	11–16 years	Mean 12.6 (5–15)
Campbell et al., 2024	USA	Retrospective cohort study	SA: Not specified	Retrospective self-report	*N* = 18,303 (34%)	Community	Not specified – Retrospectively reporting (0–18 years)	Mode 18 (ns)
Chen and Chun, 2019	Taiwan	Cross-sectional	NSSI: Behaviours in which people intentionally inflict damage to the surface of their body	Retrospective self-report	*N* = 438 (37%)	School	13–18 years	Mean Moderate NSSI: 13.5 (ns); Severe NSSI: 12.6 (ns)
Csorba et al., 2009	Hungary	Cross-sectional	SI: Including suicidal and non-suicidal self-injury	Retrospective self-report	*N* = 105	Outpatient	14–18 years	Mode 14.0 (ns)
Defayette et al., 2020	USA	Cross-sectional	SA: Not specified	Retrospective self-report	*N* = 95	Hospital inpatient	13–18 years	Mean 14.2 (7–17) Single attempt: 14.8 (ns); Multiple attempt: 13.6 (ns)
Gallegos-Santos et al., 2018	Peru	Cross-sectional	SI: The intentional injurious action that occurs in the body of the person who carries them out, causing bodily harm of low lethality and of a socially unacceptable nature, thus being a deliberate action that occurs repeatedly with the latent risk of becoming chronic	Retrospective self-report	*N* = 997 (28%)	School	13–18 years	Mean 12.6 (8–18)
García-Nieto et al., 2015	Spain	Cross-sectional	NSSI: Direct, deliberate destruction of body tissue without suicidal intent	Retrospective self-report	*N* = 239 (24%)	Outpatient	11–18 years	Mean 10.6 (ns)
Glenn and Klonsky, 2013	USA	Cross-sectional	NSSI disorder: DSM-5 criteria	Retrospective self-report	*N* = 198 (49%)	Hospital inpatient	12–18 years	Mean 12.8 (ns)
Glenn et al., 2017	USA	Prospective longitudinal	NSSI: Actions aimed at directly and deliberately injuring oneself without intent to die; SA: Actions aimed at directly and deliberately injuring oneself in which an individual has at least some intent to die during the self-injurious act	Retrospective self-report	*N* = 174	Hospital inpatient	13–18 years	Mean NSSI: 13.2 (5–17); SA: 14.3 (7–18)
Gromatsky et al., 2020	USA	Prospective longitudinal	NSSI: The direct, deliberate destruction of one’s own body tissue with the absence of the intent to die	First onset during the study period	*N* = 462 (9%)	Community	13–15 years	Mean 15.3 (ns)
Groschwitz et al., 2015	Germany	Retrospective, follow-up	NSSI: Deliberate destruction of body tissue without suicidal intent	Not specified	*N* = 52	Hospital	18–28 years – Retrospectively reporting (ns)*	Mean 13.9 (9–17) Prevailing NSSI: 13.8 (ns); Ceased NSSI: 14.0 (ns)
Hankin and Abela, 2011	USA	Prospective longitudinal	NSSI: The direct, deliberate destruction of body tissue without lethal intention	Retrospective self-report	*N* = 103 (8%)	Community	11–14 years	Mean 11.2 (ns)
Itzhaky et al., 2020	USA	Retrospective	SA: An intentional self-injurious act performed with at least some intent to die	Retrospective self-report	*N* = 43	Mixed - clinical and community	18–64 years – Retrospectively reporting (0–12 years)	Mean 9.8 (5–12)
Izadi-Mazidi et al., 2019	Iran	Cross-sectional	NSSI: Deliberate, socially unacceptable destruction of one’s own bodily tissue without suicidal intent	Retrospective self-report	*N* = 646 (28%)	School	15–18 years	Mean 14.6 (ns)
Kentopp et al., 2021	USA	Cross-sectional	NSSI: The direct and deliberate destruction of one’s body tissue without suicidal intent	Retrospective self-report	*N* = 200	Hospital inpatient	13–18 years	Early initiation (> 12): 36%; No early initiation (13–18): 59%
Kim et al., 2015	USA	Cross-sectional	NSSI: the purposeful destruction of one’s body tissue without the intent to die; SA: an action, regardless of actual resulting self-injury, completed with some intent to die	Retrospective self-report	*N* = 90	Hospital inpatient	13–17 years	Mean NSSI: 13.2 (ns); SA: 14.8 (ns)
Kostic et al., 2019	Serbia	Cross-sectional	NSSI: The deliberate, self-inflicted destruction of body tissue without suicidal intent and for purposes not socially sanctioned, including cutting, burning, biting, and scratching skin	Retrospective self-report	*N* = 50	Hospital	13–18 years	Mean 14.1 (ns)
Kumar et al., 2004	USA	Cross-sectional	Self-cutting: Intentionally cut[ting] themselves without any conscious intention of committing suicide	Retrospective self-report	*N* = 50	Hospital inpatient	13–17 years	Mean 13.5 (ns)
Liu et al., 2019	China	Longitudinal - baseline data only	SA: A ‘yes’ response to the question: ‘Have you ever in your whole life tried to kill yourself?’	Retrospective self-report	*N* = 11,831 (8%)	School	12–18 years	Mean Males: 12.3 (ns); Females: 12.6 (ns)
Mirkovic et al., 2020	France	Prospective longitudinal	SA: Self-harm behaviour with suicidal intent	Retrospective self-report	*N* = 320	Hospital	13–17 years	Mean 14.6 (ns) No relapse: 14.6 (ns); New SA: 13.9 (ns)
Morey et al., 2016	England	Cross-sectional	SH: An act with a non-fatal outcome in which an individual deliberately did one or more of the following: Initiated behaviour (for example, self-cutting, jumping from a height), which they intended to cause self-harm, Ingested a substance in excess of the prescribed or generally recognised therapeutic dose, Ingested a recreational or illicit drug that was an act that the person regarded as self-harm, Ingested a non-ingestible substance or object	Retrospective self-report	*N* = 2,000 (16%)	Community	13–18 years	Median 13.0 (ns) Mean Males: 13.5 (ns); Females: 13.0 (ns)
Muehlenkamp and Brausch, 2012	USA	Cross-sectional	NSSI: Destruction of body tissue without suicidal intent and for non-socially sanctioned purpose	Retrospective self-report	*N* = 284 (27%)	Mixed - clinical and school	Not specified*	Mean 12.9 (ns) High school: 13.3 (ns); Clinical: 12.5 (ns)
Nixon et al., 2002	Canada	Cross-sectional	Self-injurious behaviours: Any means of physically harming oneself without conscious suicidal intent	Retrospective self-report	*N* = 42	Hospital inpatient	12–18 years	Mean 12.7 (ns) Males: 15.7 (ns); Females: 12.3 (ns)
Nock and Prinstein, 2004	USA	Cross-sectional	Self-mutilation: Deliberate damage to one’s own body tissue without suicidal intent	Retrospective self-report	*N* = 108 (82%)	Hospital inpatient	12–17 years	Mean 12.8 (6–17)
Ougrin et al., 2012	England	RCT	Suicidal SH: suicide attempts and self-injurious behaviour, suicidal intent unknown; Non-suicidal SH: self-injurious behaviour, no suicidal intent	Not specified	*N* = 70	Outpatient	12–18 years	Mean Suicidal SH: 15.4 (ns); Non-suicidal SH: 13.8 (ns)
Swannell et al., 2008	Australia	Cross-sectional	SI: The deliberate destruction or alteration of body tissue without suicidal intent	Not specified	*N* = 38	Hospital inpatient	14–17 years	Mean Females: 14.0 (ns); Males: 14.5 (ns)
Szewczuk-Boguslawska et al., 2021	Poland	Cross-sectional	NSSI: not related to suicidal ideation, they lead to body tissues’ damage; SA: not specified	Retrospective self-report	*N* = 196	Centre for those at risk of social maladjustment	Not specified*	Mean SBD NSSI: 12.5 (ns); SBD SA: 13.7 (ns); non-SBD NSSI: 13.1 (ns); non-SBD SA: 13.5 (ns)
Thomassin et al., 2017	USA	Cross-sectional	NSSI: Deliberate, non-socially sanctioned tissue damage done without conscious suicidal intent	Retrospective self-report	*N* = 93 (72%)	Hospital	10–17 years	Mean 11.4 (ns)
Townsend et al., 2021	Australia	Cross-sectional	SH: Intentional self-injury or poisoning	Parent reporting perceived onset of child’s self-harm	*N* = 37	Mixed - clinical and community	12–18 years	Mean 12.7 (5–18)
Uzun Cicek et al., 2022	Turkey	Cross-sectional	NSSI: Intentional and socially unacceptable harm to oneself, regardless of suicidal intent	Not specified	*N* = 96	Outpatient	12–16 years	Mean 13.1 (ns)
Venta et al., 2012	USA	Cross-sectional	SA: Not specified	Retrospective self-report	*N* = 106	Hospital inpatient	12–17 years	Mean 14.0 (ns) Non-BPD: 14.2 (ns); BPD: 13.4 (ns)
Wang B et al., 2016	China	Prospective longitudinal	NSSI: The deliberate, direct, and socially unacceptable destruction of one’s body tissue without conscious suicidal intent	Not specified	*N* = 3,381 (26%)	School	13–17 years	Mean 11.8 (13–17)
Wang H et al., 2020	China	Prospective longitudinal	NSSI: The intentional damage to one’s body tissue (e.g., scraping the skin and self-battery) without suicidal intent	First onset during the study period	*N* = 938 (16%)	School	12–16 years	Mean 13.4 (ns)
Wang P et al., 2022	Spain	Cross-sectional	NSSI: not specified; SA: not specified	Not specified	*N* = 39	Hospital inpatient	12–17 years	NSSI: 12.9 (ns); SA: 13.6 (ns)
Zetterqvist et al., 2013	Sweden	Cross-sectional	NSSI: the deliberate, self-inflicted destruction of body tissue without suicidal intent; SA: a potentially self-injurious behaviour in which there is some intent to die from the behaviour	Retrospective self-report	*N* = 2,964 (6%)	School	15–17 years	NSSI: 13.4 (ns); SA: 13.8 (ns)
*NS* not specified, *RCT* randomised controlled trial, *SA* suicide attempt, *NSSI* non-suicidal self-injury, *SI* self-injury, *SH* self-harm, *DSM-5* Diagnostic and Statistical Manual of Mental Disorders, Fifth Edition;* BPD* borderline personality disorder, *SBD* suicidal behaviour disorder, *these studies were included as they included samples generally thought not to exceed 18 years of age; ^included due to the ability to separate between ages of onset under and over 18 years of age								

The quality assessment (QuADS) highlighted that most studies scored highly in the appropriateness of the study design and tools to address the research objectives, clearly describing the research setting and target population, and making clear aims statements. However, few studies scored well in discussing the appropriateness of the study sample for addressing the research aims, providing a rationale for the data collection tools, and justifying the choice of analytical methods (see Supplementary file 4 for the full breakdown of scores assigned).

### Age of onset of self-harm overview

**Fig. 3:**
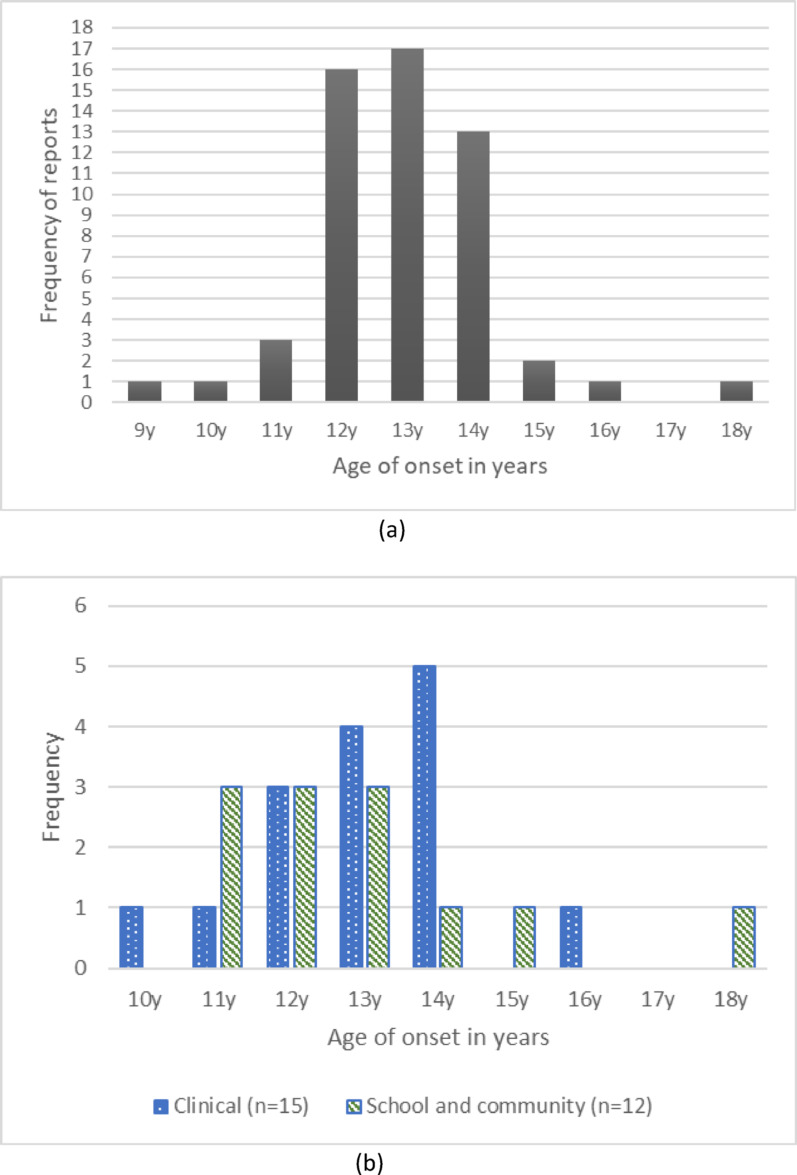
The distribution of age of onset reports **a **ages of onset reported in 42 studies **b s**chool and community vs clinical samples (n=12 studies)

Age of onset of self-harm was reported in the form of a mean (36 studies), median (three studies [9, 57, 59], mode (two studies [31, 71]), and was grouped as ‘early initiation’ and ‘no early initiation’ (one study [43]). Twenty studies reported age of onset according to certain subgroups [33, 39, 40, 44, 45, 48, 49, 52, 53, 55, 56, 58–61, 63, 65, 67, 69, 71], 11 of which reported according to subgroups only. There were 55 independent ages of onset reported across the included studies, this was inclusive of studies reporting on their entire sample in addition to studies reporting on subgroups separately. The range of the mean ages of onset was 9 years to 18 years for entire samples. The modal onset age was 13 years (*n* = 17), followed by 12 years (*n* = 16), and 14 years (*n* = 13); the remaining ages (9, 10, 11, 15, 16, and 18 years) occurred less than three times each (Fig. 3a). The distribution of onset age within studies including clinical samples (those given a psychiatric diagnosis) versus community or school samples are presented in Fig. 3b for studies reporting on the entire sample. Five and eight studies report on subgroups only in school or community and clinical samples respectively. The distribution of onset age in school and community settings is skewed to younger ages, whereas onset age for those given a psychiatric diagnosis appears to be slightly older. Kentopp et al. [43] reported early initiation of self-harm (12 years or younger) in 36% and non-early initiation (13–18 years) in 59% of the sample.

In studies reporting age of onset for the entire sample (*n* = 30), 60.0% did not provide a range of onset age, this was also the case for 17 of the 20 studies reporting according to sample characteristics and categories.

**Fig. 4:**
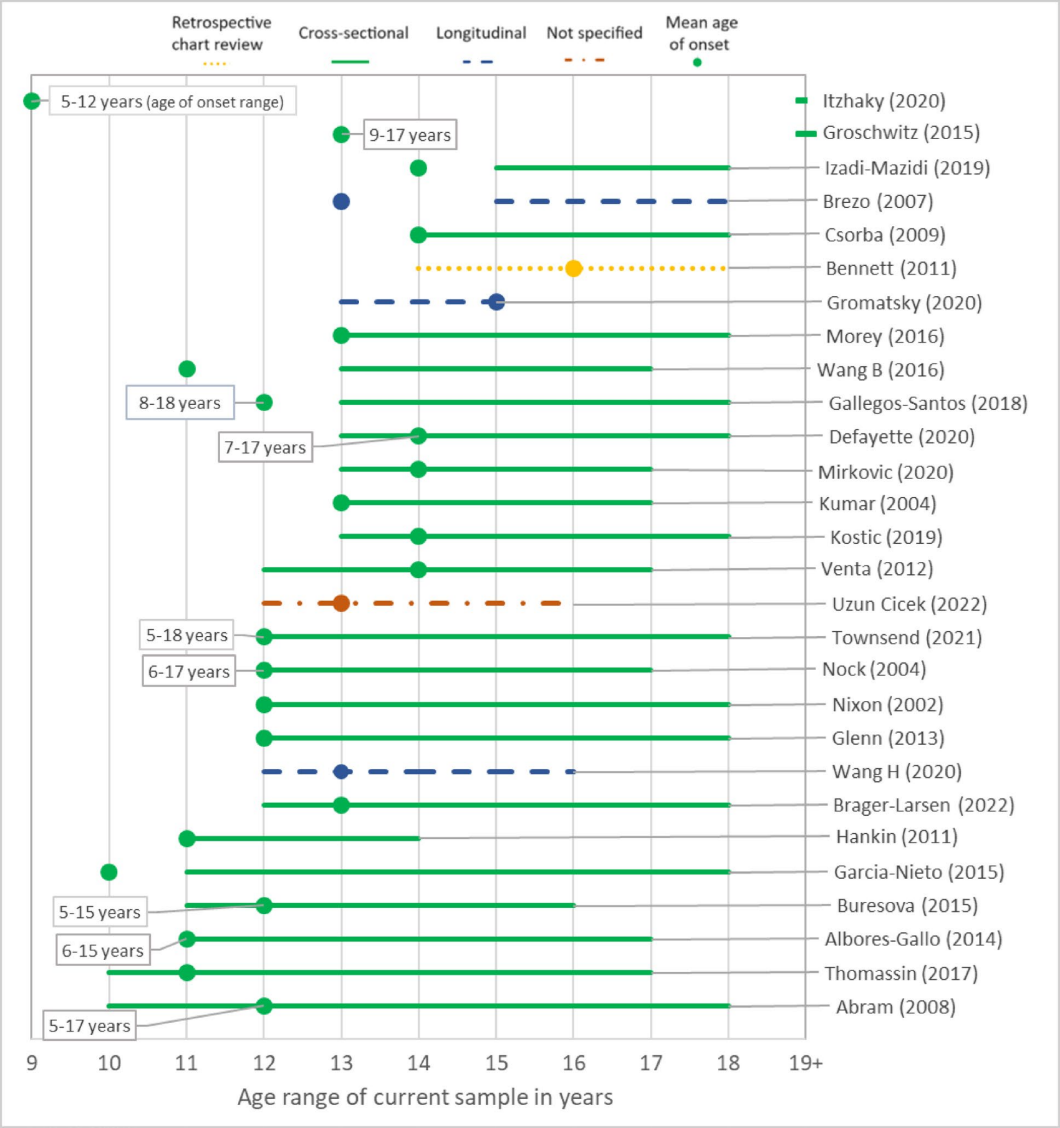
Age of onset with reference to sample age range and study design

The age range of each study sample was found to be important in understanding the age of onset data reported. Figure 4 illustrates 28 studies where age of onset (including sample age range) was reported as a measure of central tendency for the entire sample. Overall, there was a trend towards studies reporting earlier age of onset of self-harm when younger ages were included in the sample.

It is important to note that the study conducted by Itzhaky et al. [42] collected age of first suicide attempt across the lifespan and then categorised the ages into groups: childhood (under 12 years), adolescence (13–19 years), and adulthood groups (20 years and older) and generated means for these groups. Given the eligibility criteria of this review, only the childhood mean age of first attempt was extracted, as the adolescent group included individuals aged up to 19 years.

### Definitions, research methods, operationalisations

Eleven different terms for self-harm were investigated. As six studies investigated two types of self-harm, there were a total of 48 terms for which onset was reported. The terms used by each study are presented in Table 3. The range of mean age of onset for NSSI and suicide attempts was 10 to 15 years and 9 to 18 years respectively.

**Table 3:** Self-harm terms investigated by the included studies

Self-harm term	%	Studies
Non-suicidal self-injury (NSSI)	41.7%	Albores-Gallo., 2014; Andrews et al., 2013; Chen & Chun, 2019; Garcia-Nieto et al., 2015; Glenn et al., 2017; Gromatsky et al., 2020; Groschwitz et al., 2015; Hankin & Abela, 2011; Izadi-Mazidi et al., 2019; Kentopp et al., 2021; Kim et al., 2015; Kostic et al., 2019; Muehlenkamp & Brausch, 2012; Szewczuk-Boguslawska et al., 2021; Thomassin et al., 2017; Uzun Cicek et al., 2023; Wang et al., 2017; Wang et al., 2020; Wang et al., 2022; Zetterqvist et al., 2013
Suicide attempt	29.2%	Abram et al., 2008; Aouidad et al., 2020; Brezo et al., 2007; Campbell et al., 2024; Defayette et al., 2020; Glenn et al., 2017; Itzhaky et al., 2020; Kim et al., 2015; Liu et al., 2019; Mirkovic et al., 2020; Szewczuk-Boguslawska et al., 2021; Venta et al., 2012; WANG et al., 2022; Zetterqvist et al., 2013
Self-harm	8.3%	Brager-Larsen et al., 2022; Buresova et al., 2015; Morey et al., 2017; Townsend et al., 2021
Self-injury	6.3%	Csorba et al., 2009; Gallegos Santos et al., 2018; Swannell et al., 2008
Suicidal self-harm and non-suicidal self-harm	2.1%	Ougrin et al., 2012
Self-mutilative behaviour	2.1%	Nock and Prinstein, 2004
Self-injurious behaviours	2.1%	Nixon et al., 2002
Self-embedding	2.1%	Bennett et al., 2011
Non-suicidal self-injury disorder	2.1%	Glenn and Klonsky, 2013
Self-cutting	2.1%	Kumar et al., 2004

Definitions were provided for 81.3% of the 48 terms. Of these, 84.6% referred to the presence or absence of suicidal intent. The most common definitions included the element of damage to body tissue without suicidal intent (48.7%), and this definition was applied mostly to NSSI [32–34, 41, 43–45, 47, 50–52, 63, 64, 67, 68], but also to self-harm [62], self-cutting [36], self-mutilative behaviour [46], and self-injury [53]. The age of onset of self-harm in studies employing this definition ranged from 10 to 15 years. Including some level of suicidal intent was applied to 17.9% of terms, all of which were suicide attempts or suicidal self-harm [33, 40, 42, 44, 49, 56, 60]. The age of onset of self-harm defined as such ranged from 9 to 15 years. In eight instances suicide attempts were not defined by study authors [37, 39, 48, 55, 57, 61, 67, 71]. The remaining definitions were mixed and used to define a variety of terms.

**Fig. 5:**
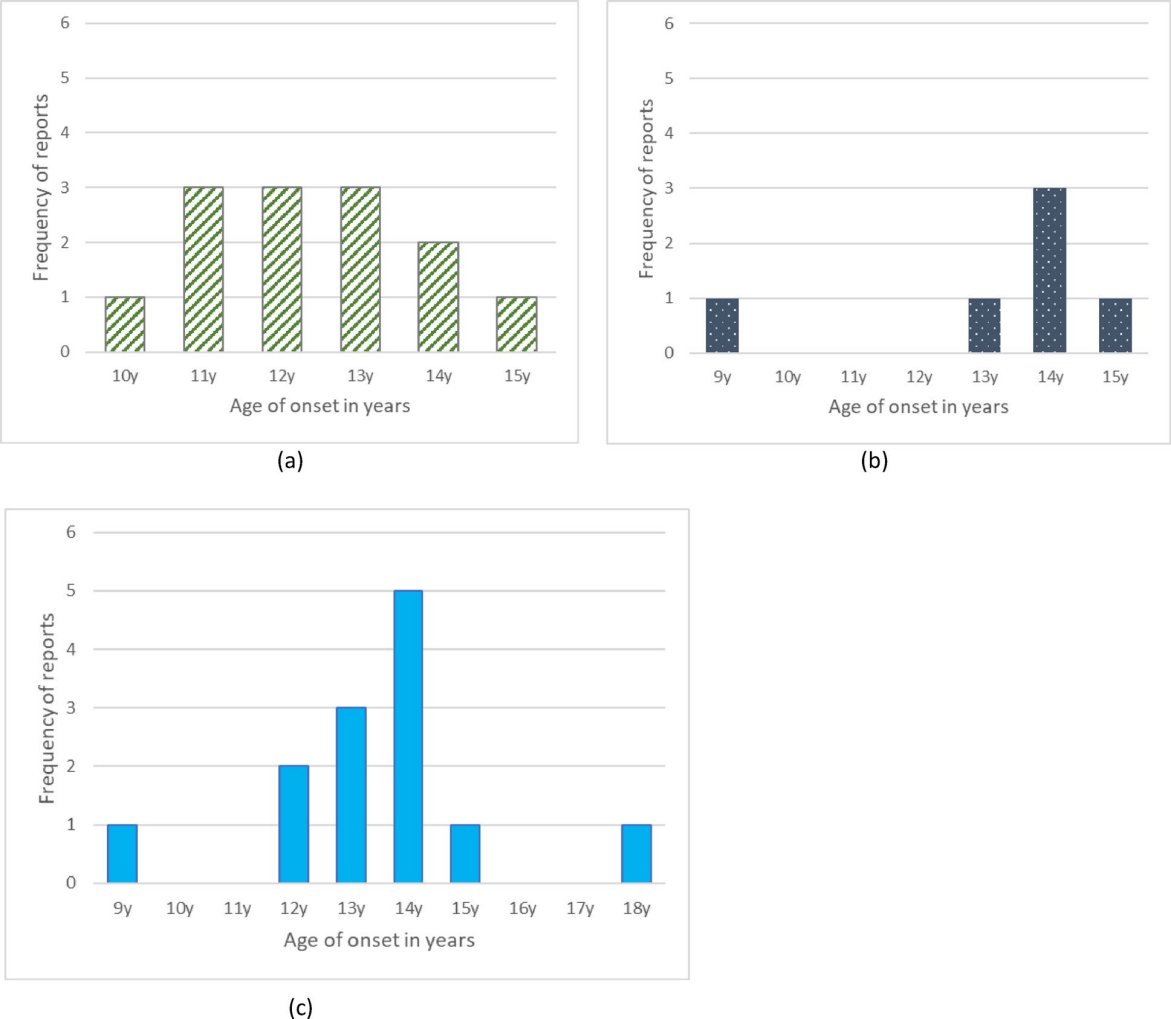
Age of onset reports per definition **a **tissue damage without suicidal intent (n = 13 studies) **b **some suicidal intent (n = 6 studies) **c **some suicidal intent + suicide attempts not defined (n = 13 studies)

The distribution of onset age for the most common definitions is illustrated in Fig. 5. Figure 5a shows the distribution of the age of onset of self-harm defined as tissue damage without suicidal intent, with the mode spanning 11–13 years. Figure 5b includes self-harm defined as having some suicidal intent, Fig. 5c further includes those specifically investigating suicide attempts without defining it. The distribution for suicide attempts widens, however 14 years is the mode in both groups.

Age of onset information was collected in a cross-sectional design in 81.0% of studies [9, 31–37, 39, 40, 42–50, 52, 54–56, 58, 59, 62–69, 71]. Three studies collected age of onset longitudinally [41, 51, 57], and one used retrospective chart review [38].

The operationalisation of onset was explicitly reported in 33.3% of studies and not possible to discern in 19.0%. In some instances the operationalisation of onset could be discerned. For example, if a cross-sectional study used a tool that asked about the age at which the participant self-harmed for the first time, the authors assumed that participants were retrospectively reporting. This was the case for 19 studies (45.2% [9, 31, 33–35, 37, 39, 40, 42, 44, 52, 58, 62, 64–66, 68, 69, 71]), while 11 studies (25.6% [32, 36, 43, 45–49, 56, 59, 67]) explicitly stated that retrospective report was used to operationalise onset. As such, retrospective reporting on first experience was the majority. Over one third of studies (38.1%) did not report the methodological tool (e.g., interview, questionnaire) used to collect age of onset data.

### Age of onset subgroups

This section briefly describes the 20 studies which reported age of onset across sample characteristics and study outcomes. As outlined in Table 4, five studies reported for males and females [49, 53, 58, 59, 65], five reported on both NSSI and suicide attempts [33, 40, 44, 60, 61], and five reported on trajectory or severity of self-harm [39, 52, 56, 63, 69]. The studies grouped as ‘trajectory of self-harm’ used a mixture of retrospective, prospective, and cross-sectional designs, so the resulting ages of onset should be interpreted with caution. Three studies reported across characteristics of psychiatric diagnoses, one compared a clinical inpatient and a school sample, one created groups according to severity using a latent class analysis, and one compared groups of transgender youth across levels of familial support.

Studies reporting on gender investigated suicide attempts, self-harm, NSSI, and self-injury. In all studies except one, the age of onset was within the same year for males and females; two of these studies took place in a clinical setting. Studies examining non-suicidal and suicidal self-harm generally reported an earlier age of onset for non-suicidal self-harm, four of these studies were conducted in a clinical setting. The age of onset was generally younger for those who had continuing or severe self-harm versus ceased or moderate self-harm and in those with multiple versus single suicide attempts; three of these studies investigated clinical samples.

**Table 4:** Self-harm age of onset in subgroups

	Gender	
*First author (year)*	*Males (years)*	*Females (years)*
Albores-Gallo (2014)	11.9	11.9
Liu (2019)	12.3	12.6
Morey (2016)	13.5	13.0
Nixon (2002)	15.7	12.3
Swannell (2008)	14.5	14.0
	*Type of self-harm*	
*First author (year)*	*NSSI (years)*	*Suicide attempt (years)*
Glenn (2017)	13.2	14.3
Kim (2015)	13.2	14.8
Ougrin (2012)	13.8 (NSSH)	15.4 (SSH)
Wang P (2022)	12.9	13.6
Zetterqvist (2013)	13.4	13.8
	*Trajectory of self-harm*	
*First author (year)*	*Stopped (years)*	*Continuing (years)*
Andrews (2013)	12.9	12.3
Chen (2019)	13.5 (moderate NSSI)	12.6 (severe NSSI)
Defayette (2020)	14.8 (single attempt)	13.6 (multiple attempts)
Groschwitz (2015b)	14.0	13.8
Mirkovic (2020)	14.6	13.9

*NSSH nonsuicidal self-harm, *SSH* suicidal self-harm

The three studies reporting onset age in those who had been given psychiatric diagnoses versus those who had not varied somewhat. Two investigated borderline personality disorder (BPD), one compared age of onset between those with and without BPD who had a single suicide attempt and those with multiple suicide attempts (all had a mean age of 14 years) [55], and the other compared age of first suicide attempt in those with BPD, (13 years) and without BPD (14 years) [48]. The third study [67] examined onset age of NSSI and suicide attempts in people who did and did not meet criteria for suicidal behaviour disorder (a disorder proposed for further consideration in the DSM-5 [72]), all groups had an onset age of 13 years, except the group who met suicidal behaviour disorder criteria and experienced NSSI (12 years). One study compared the onset age of NSSI in a clinical inpatient and school sample (12 years and 13 years respectively) [45]. Finally, a single study focusing on familial support of transgender youth reported a modal age of 18 years at first suicide attempt across supportive, adverse, and neutral families [71].

## Discussion

The aim of this scoping review was to capture age of onset of self-harm in children and adolescents with respect to the relevant contexts, operationalisations, and research methods used. Most studies were conducted in hospital, school, or community settings and operationalisation of onset was not often explicitly described; however, the use of retrospective reporting was in the majority. Often, self-harm was conceptualised dichotomously based on the level of suicidal intent with variations in the distribution of age of onset visible within this dichotomy. A sizable minority did not report the methods used to capture age of onset data. Generally there was a trend towards reporting an earlier age of onset of self-harm where study samples included younger ages. The overall modal age of onset estimate was 13 years, with most of the reported measures of central tendency falling between 12 and 14 years of age.

### Findings in context

Two prior systematic reviews report an estimate for self-harm age of onset. Gillies et al. [18] included all categories of community-occurring self-harm in those aged between 12 and 18 years in their review, with 12.81 years being the onset age estimate based on 14 studies (*n* = 22,031 participants). Cipriano et al. [17] investigated non-suicidal self-injury in samples of all ages (range of mean ages reported, 11.6 to 55.5 years), reporting that self-harm onset most often occurs between 12 and 14 years, with some studies reporting the behaviour in those under 12 years. The age of onset reported in the current review is similar. However, this scoping review represents the first attempt, to our knowledge, to gather the age of onset of self-harm from studies, taking account of how onset and self-harm were operationalised. In doing so, this review highlights some important characteristics of the literature.

Most studies in this review specified self-harm as either non-suicidal, which was the most prevalent, or as suicide attempts or self-harm with some level of suicidal intent. The age of onset distribution appeared to be different across the two categories, non-suicidal self-harm was slightly younger than the onset of suicide attempts. This may indicate that there are differing motives of self-harm at different times during adolescence and there may be different pathways and contributory factors to NSSI onset and first suicide attempt. Alternatively, it could signal an escalation in self-harm severity for those who engage in both NSSI and suicide attempts (e.g., the Gateway Theory [73]); although, such an escalation in severity has previously been reported to occur later, during early adulthood [74].

There were several noteworthy findings related to study design, onset operationalisation, and methods or tools used to collect data. Almost all studies used a cross-sectional design to gather age of onset data and thus many participants in these studies used retrospective report in identifying their first act. Prospective studies aimed at capturing onset in young people who had not yet self-harmed were rare. Retrospective report is subject to recall bias. A previous review found inconsistent reporting of lifetime suicide attempts was very common, and approximately one third will not report self-injurious thoughts and behaviours after having previously disclosed them [75]. This review included both adolescent and adult samples while the majority of studies in the current review focused on child and adolescent samples, limiting the timeframe participants reflected on. However, this could still be a matter of years as an 18-year-old may be recalling their first experience at 12 or 13 years. Figure 4 highlights that in many of the studies, the mean age of onset was the youngest age of the sample or earlier. Considering the discrepancy in reporting highlighted by Klimes-Dougan et al., this finding may point to onset occurring even be earlier, yet not recalled [75]. Prospective studies which begin prior to the most common estimates of age of onset, with shorter intervals between follow-up data capture, would enhance the robustness of the evidence.

What also came out in this review was the use of different terms, such as self-harm or NSSI for an act similarly defined. This heterogeneity in nomenclature is a feature of self-harm research, with diverging opinions held on what terms (e.g., a suicide attempt, self-harm, NSSI among others) describe acts that cause harm but the intent is to die, not to die, or unknown [76]. It extends to epidemiological evidence, with studies in similar settings reporting on incident rates of ‘self-harm’, ‘suicide attempts’, and ‘parasuicide’ [19] and reporting prevalence rates for ‘deliberate self-harm’ and ‘NSSI’, with the former including acts with suicidal intent [18]. To promote consensus, the International Study of Definitions of English-Language Terms for Suicidal Behaviours proposed recommended nomenclature to advance toward a universal classification of suicidal behaviour. A shared understanding of self-harm is required to meaningfully progress in understanding age of onset.

More than one third of studies did not report their methods or tools used to capture age of onset data. While this likely occurred due to onset often not being an objective of the study, it has implications for the design and implementation of prevention and intervention strategies. The variability in explicit reporting of operationalisations and tools obscures an accurate and nuanced understanding of the age of onset of self-harm, which has been highlighted as key for orienting the focus of prevention and intervention strategies for mental distress more broadly [77].

### Strengths and limitations

The search strategy is a strength of the review; it was created in consultation with and validated by a librarian. Additionally, the included studies underwent quality assessment. While this is not required for a scoping review it can improve the usefulness of the scoping review findings for practice and future research [30].

There are some limitations to discuss. As highlighted by the findings, just under two thirds of studies reported age of onset without mention in the aims, objectives, or hypotheses. This leads us to believe that there may be studies which report age of onset but were not detected by the search strategy or screening process. To overcome this limitation, the eligibility criteria during the title and abstract phase were less stringent and studies indirectly referring to onset or development of self-harm were forwarded for full text screening.

Furthermore, the eligibility criteria were amended during the review process to specifically include studies that report age of onset as a measure of central tendency. A measure of central tendency was chosen as it was the most common method of reporting age of onset in the screened records at that point. However, it resulted in studies which used other methods to report age of onset of self-harm being excluded [78, 79]. The age of onset reported in both these studies is slightly older than the modal age of onset from the current review, with peak hazard for beginning self-harm being 14 to 15 years [79] and 15 years [78]. Finally, this review included studies of young people aged 18 years and younger, making the findings generalisable to this age group only.

### Relevance to clinical practice

The results of this review suggest that, consistent with prior reviews, the average age of self-harm onset falls between 12 and 14 years. The distribution of onset age of self-harm in community samples favoured a younger age compared to clinical samples. It is possible that those in clinical samples began self-harm earlier than is detected by services. Many adolescents who attend hospital do not present to services for prior self-harm [80], possibly reflecting the delay seen in help-seeking for mental distress more generally [81]. This could be mapped onto the iceberg of suicide and self-harm. This model proposes that suicide is the most rare and visible outcome (iceberg tip), self-harm that presents to hospital is more common (large part still above water), and self-harm that occurs in the community is the most common but remains largely hidden (largest part of the iceberg submerged underwater) [82, 83]. It is possible that some young people occupy both the community occurring self-harm and presenting to services levels of the iceberg at different times.

Finally, professionals in clinical practice should be cognisant of limitations in the literature such as the heterogeneity in nomenclature, common use of retrospective reports, and the lack of transparency in reporting the tools used to capture this data. These methodological features obscure a clear understanding of self-harm age of onset.

### Future directions

This review identified a dearth of research using methods other than retrospective self-report to capture age of onset of self-harm, the limitations of which have been discussed. Future research could utilise self-harm surveillance systems or registers (e.g., National Self-harm Registry Ireland [1], Multicentre Study of Self-harm in England [84]) as their universal coverage may overcome the limitations of retrospective report [85] and enable the provision of more robust estimates of self-harm age of onset.

Furthermore, the differences between the onset age for clinical and community samples, and NSSI and suicide attempts are of interest. It would be worthwhile for future studies to prospectively explore trajectories of self-harm behaviour, including escalation, cessation, and help-seeking, with a sufficient sample size [86]. This would allow the identification of key points in self-harm trajectories where intervention might be most appropriate.

Finally, while examining changes in onset age over time was not an objective of this review, a preprint has recently reported that the age of onset of self-harm has decreased for younger generations [87]. This emerging research, alongside other research reporting increasing rates in younger age groups [6, 8] further underscores the need for high quality studies investigating the age of onset of self-harm.

## Conclusion

This scoping review highlights the age range of 12 to 14 years as the typical onset period for self-harm. Studies reporting on subgroups found a similar age of onset for males and females, but a lower age of onset for those who engaged in NSSI versus suicide attempts and those who repeat self-harm compared to those who do not. There is considerable scope for improving methods to estimate age of onset of self-harm. Recent research suggests a decreasing age of onset. The link between earlier onset and a more severe self-harm trajectory, coupled with the importance of timely prevention and intervention places an urgency on obtaining an accurate understanding of age of onset of self-harm. Timely prevention and intervention is paramount to reduce distress and self-harm, making it critically important to obtain an accurate understanding of age of onset of self-harm.

## Supplementary Information

Supplementary Material 1.

Supplementary Material 2.

Supplementary Material 3.

Supplementary Material 4.

## Data Availability

The data that support the findings of this review are available from the authors upon reasonable request..

## Abbreviations

SA: Suicide attempt
NSSI: Non-suicidal self-injury
SI: Self-injury
SH: Self-harm
SBD: Suicidal behaviour disorder
OSF: Open science framework
QuADS: Quality assessment with diverse studies
RCT: Randomised controlled trial
BPD: Borderline personality disorder
DSM-5: Diagnostic and Statistical Manual of Mental Disorders: Fifth Edition
