# Supplementary material for: Age of onset of self-harm in children and adolescents: a scoping review
Source: Child Adolesc Psychiatry Ment Health. 2025 Nov 19;19:128. doi: 10.1186/s13034-025-00982-6 (PMC12629077; doi:10.1186/s13034-025-00982-6)
Supplement: Supplementary file 2 — Supplementary Material 2. [file 13034_2025_982_MOESM2_ESM.docx]

# APPENDIX 2

# Amendments to eligibility criteria:

**Sample first self-harmed at 17 years or younger/ Age of first self-harm act was reported to be in adulthood (18 years and above)**

Reasons for amending this:

- The sample from which the age of first act is reported varied in age, age of first act could be 13 years for a sample of 10-18 year olds and a sample of 18-30 year olds, it was agreed with the authorship team that such samples are very different.
- The age of first act reported could come from a sample which spanned under and over 18 years

Reason for changing it to current criterion:

- The focus of the study is on children and adolescents and thus made the review more coherent
- Studies reporting on samples aged up to and including 18 years were not wholly different from samples up to and including 17 years in terms of the population of interest, in most if not all cases, both types of sample were referred to as adolescents

**Age of onset reported**

Reasons for amending this:

- The criterion was not specific enough to make decisions on the eligibility of some records, for example, some records reported on first acts for age groups which spanned above and below 18 years
- Records reported age of first act in a variety of ways, for example, providing a range (12-14 years), reporting the age of each participant

Reason for changing it to current criterion:

- Mean, median, and mode are commonly reported measures in studies and were thought to be the most inclusive way to create a specific eligibility criterion

**Study examines stereotypic self-harm behaviours more common in intellectual disabilities and autism (e.g., intense, repetitive, rhythmic behaviours such as eyeball pressing and head banging)**

Reasons for amending this:

- The review identified records which focused on self-harm driven by a biological need, an illness unrelated to self-harm, a prior injury which were deemed as outside the scope of the review

**Study protocols, editorials, letters to the editor, case reports, case series**

Reasons for amending this:

- Reviews of the literature were left out of the eligibility criteria in error, however this criterion was described in the text of the protocol

**New criteria:**

- There was no guidance as to what to do with records which included samples with mixed ages. Therefore, it was decided that if a study reports on age of first act separately for any subsamples aged under 19 years and 19 years and older, the information available on subsamples under 19 years would be extracted.
- There was no guidance on what to do with records which reported an age of first act that was not based on a sample of adolescents, i.e., professional opinion. It was decided that these would be excluded because the estimate was not based on any data collected from young people.
- There was a small number of papers that did not report on self-harm behaviours and other phenomena separately, i.e., accidental overdose, thoughts of self-harm. As we are explicitly interested in the behaviour of self-harm, these were excluded.

# Amendments in relation to citation searching:

In the protocol we stated that we would search articles cited by the included studies and articles that cite the included studies. Having logged in using an author’s institutional email address, we used Scopus’ document search (<https://www-scopus-com.ucc.idm.oclc.org/search/form.uri?zone=TopNavBar&origin=recordpage&display=basic#basic>) to estimate the numbers of records this would entail screening. In combination, it exceeded 4,000 additional articles to be screened. Backward searching alone resulted in 1,860 records to be screened which was deemed feasible to complete.

# Amendments in relation to the data extraction form:

Items in italics were added during the pilot process

| **Extraction items** | **Reason for change** |
| --- | --- |
| 1. Primary author | No changes |
| 1. Year of publication |  |
| 1. *Title* | Not included in the protocol in error |
| 1. *Publication* |  |
| 1. *Evidence source type* |  |
| 1. *Language of article* |  |
| 1. Funding source(s) | No changes |
| 1. Geographic setting(s) of the study |  |
| 1. Study design |  |
| 1. Study period |  |
| *10a. Length of follow-up* | Informative to know, in the case of longitudinal studies, the length of the follow-up period |
| *10b. Number of times data were collected* | Informative to know, in the case of longitudinal studies, how many times data were collected over follow-up |
| *10c. Prospective – onset data collection time* | This item arose as some studies collected onset information at baseline, while others measured onset over follow-up which is important to distinguish |
| 1. *Population (age group)* | This item was added to capture how the study described young people (eg. juveniles, adolescents) |
| *12. Population (clinical status)* | This item was added to easily denote whether the sample was clinical or not |
| *12a. Diagnostic tool/classification* | This item was added to capture which standardised tool or diagnostic classification was used in clinical sample to provide descriptive information |
| *13. Recruitment strategy* | This information was deemed relevant to understand the boundaries of the studies that are reporting age of onset of self-harm |
| *13a. Exclusion reasons* |  |
| 14. Sample size | No changes |
| *14a. Self-harm is a subset* | This item was added because in some papers, the total sample was not those who had self-harmed or reported an age of onset |
| *14b. Age of onset reports split as per study defined categories* | This item was added as it allows the streamlining of collecting age of onset information when it is not reported for the entire sample or is reported according to specified study outcomes or sample characteristics in addition to the entire sample |
| *14c. Categories of onset defined* | To streamline extraction and analysis process |
| *14d. Number in the subset(s)* |  |
| 1. *Study setting* | To add descriptive details which may be relevant to interpreting the age of onset being reported |
| 1. First act included in study aims | No changes |
| 1. Self-harm term used |  |
| 1. Self-harm definition used |  |
| 1. First self-harm act operationalisation used |  |
| 1. Method used to determine age of first act |  |
| 1. Method used to assess self-harm behaviour |  |
| 1. Sample characteristics |  |
| 22a. Age of overall sample |  |
| *22b. Age range of sample* | Adds important descriptive information about the sample, moreover the age range of the sample (in the case of longitudinal studies for example) will impact on the age of onset captured |
| *22c. Retrospectively reporting* | This item streamlined the analysis process by denoting whether the sample included those over 18 years who were retrospectively reporting on adolescence |
| *22d. Retrospective time period* | This item was added for the reason being that the age range of focus influences the age of onset reported |
| 22e. Gender | No changes |
| *22f. Diagnoses/ symptoms* | Introduced instead of “contact with mental health services”, this was captured in study setting; the new item captured more detailed clinical information |
| 22g. Method of first self-harm act | No changes |
| 1. *Measure of central tendency used* | To streamline extraction and analysis process |
| 1. *Age of first act (entire sample/first act subset)* |  |
| *24a. Entire: measure of spread* |  |
| *24b. Entire: age range of first act* |  |
| 25. Subsamples: name of category 1 |  |
| 25a. Subsamples: Age of first act – category 1 |  |
| 25b. Subsamples: measure of spread - category 1 |  |
| 25c. Subsamples: age range of first act - category 1 |  |
| 26. Subsamples: name of category 2 |  |
| *26a. Subsamples: Age of first act – category 2* |  |
| *26b. Subsamples: measure of spread - category 2* |  |
| *26c. Subsamples: age range of first act - category 2* |  |
| *27. Subsamples: name of category 3* |  |
| *27a. Subsamples: Age of first act – category 3* |  |
| *27b. Subsamples: measure of spread - category 3* |  |
| *27c. Subsamples: age range of first act - category 3* |  |
| *28. Subsamples: name of category 4* |  |
| *28a. Subsamples: Age of first act – category 4* |  |
| *28b. Subsamples: measure of spread - category 4* |  |
| *28c. Subsamples: age range of first act - category 4* |  |
| *29. Multiple measures of central tendency* |  |
| *29a. Additional age of onset information 1* |  |
| *29b. Additional age of onset information 2* |  |
